# Supplementary material for: Predictive Value of Soluble PD-1, PD-L1, VEGFA, CD40 Ligand and CD44 for Nivolumab Therapy in Advanced Non-Small Cell Lung Cancer: A Case-Control Study
Source: Cancers (Basel). 2020 Feb 18;12(2):473. doi: 10.3390/cancers12020473 (PMC7072584; doi:10.3390/cancers12020473)
Supplement: Supplementary file 1 [file cancers-12-00473-s001.zip › cancers-707774-supplementary figures and tables/Supp table 1 sCombo.docx]

**Supplementary Table S1:** Plasma biomarkers’ concentrations at baseline and day 28 (after two cycles of nivolumab).

| **Biomarker** | **Baseline**  **median [IQR]** | **Day 28**  **median [IQR]** | **P*** |
| --- | --- | --- | --- |
| **NIVOLUMAB GROUP** | | | |
| **sPD-1 (ng/ml)** | 0.07 (0.03–0.18) | 0.07 (0.03–0.20) | 0.524 |
| **sPD-L1(ng/mL)** | 0.16 (0.03–0.44) | 0.13 (0.03–0.38) | 0.293 |
| **VEGFA** (**pg/mL)** | 97 (56–179) | 103 (61–171) | 0.809 |
| **sCD44** (**ng/mL)** | 3.3 (2.1–6.6) | 4.31 (2.55–5.88) | 0.149 |
| **sCD40L** (**pg/mL)** | 80 (3–428) | 155 (8–342) | 0.759 |
| **EGFR-MUTATED GROUP** | | | |
| **sPD-1 (ng/mL)** | 0.11 (0.07–0.21) | – |  |
| **sPD-L1 (ng/mL)** | 1.50 (0.70–2.22) | – |  |

IQR: interquartile range; VEGFA: Vascular Endothelial Growth Factor A. *A paired Wilcoxon test was used to compare plasma biomarker levels at baseline and day 28.
