# Supplementary material for: Predictive Value of Soluble PD-1, PD-L1, VEGFA, CD40 Ligand and CD44 for Nivolumab Therapy in Advanced Non-Small Cell Lung Cancer: A Case-Control Study
Source: Cancers (Basel). 2020 Feb 18;12(2):473. doi: 10.3390/cancers12020473 (PMC7072584; doi:10.3390/cancers12020473)
Supplement: Supplementary file 1 [file cancers-12-00473-s001.zip › cancers-707774-supplementary figures and tables/Supp table 2 sCombo.docx]

**Supplementary Table S2: Comparison of biomarkers’ concentrations in nivolumab group and their correlation to CRP, NLRand nivolumab concentration at day 28.**

|  | **sPD-1 D0** | **sPD-L1D0** | **VEGFA D0** | **sCD44 D0** | **sCD40L D0** | **PD-L1 TC** | **CRP D0** | **NLR D0** | **sPD-1 D28** | **sPD-L1 D28** | **VEGFA D28** | **sCD44 D28** | **sCD40L D28** | **Nivolumab** | **CRP D28** | **NLR D28** |
| --- | --- | --- | --- | --- | --- | --- | --- | --- | --- | --- | --- | --- | --- | --- | --- | --- |
| **sPD-1 D0** | 1 |  |  |  |  |  |  |  |  |  |  |  |  |  |  |  |
| **sPD-L1D0** | 0.27 | 1 |  |  |  |  |  |  |  |  |  |  |  |  |  |  |
| **VEGFA D0** | 0.14 | 0.08 | 1 |  |  |  |  |  |  |  |  |  |  |  |  |  |
| **sCD44 D0** | -0.06 | -0.16 | 0.17 | 1 |  |  |  |  |  |  |  |  |  |  |  |  |
| **sCD40L D0** | 0.34 | 0.03 | 0.23 | 0.29 | 1 |  |  |  |  |  |  |  |  |  |  |  |
| **PD-L1 TC** | 0.1 | 0.14 | 0.08 | -0.12 | -0.09 | 1 |  |  |  |  |  |  |  |  |  |  |
| **CRP D0** | -0.03 | 0.11 | 0.02 | -0.24 | -0.47 | -0.13 | 1 |  |  |  |  |  |  |  |  |  |
| **NLR C0** | -0.09 | 0.1 | 0.21 | -0.07 | 0.02 | 0.11 | 0.11 | 1 |  |  |  |  |  |  |  |  |
| **sPD-1 D28** | 0.85 | 0.29 | 0.02 | -0.02 | 0.2 | 0.11 | 0.01 | -0.14 | 1 |  |  |  |  |  |  |  |
| **sPD-L1 D28** | 0.24 | 0.69 | 0.19 | 0.16 | 0.21 | 0.05 | -0.01 | -0.06 | 0.32 | 1 |  |  |  |  |  |  |
| **VEGFA D28** | -0.25 | -0.11 | 0.61 | 0.15 | 0.06 | 0.03 | 0.18 | 0.04 | -0.31 | 0.16 | 1 |  |  |  |  |  |
| **sCD44 D28** | 0.18 | -0.13 | -0.08 | 0.4 | -0.12 | -0.17 | 0.11 | -0.16 | 0.25 | 0.04 | -0.08 | 1 |  |  |  |  |
| **sCD40L D28** | 0.11 | 0.11 | 0.37 | 0.15 | 0.56 | -0.17 | -0.2 | 0.05 | 0.05 | 0.4 | 0.36 | 0.06 | 1 |  |  |  |
| **Nivolumab** | 0.05 | -0.12 | 0.05 | 0.2 | 0.3 | 0.11 | -0.41 | 0.01 | -0.12 | -0.16 | -0.03 | -0.18 | -0.03 | 1 |  |  |
| **CRP D28** | -0.09 | 0.09 | 0.15 | -0.06 | -0.32 | -0.1 | 0.78 | 0.3 | -0.06 | 0.02 | 0.38 | 0.13 | -0.13 | -0.31 | 1 |  |
| **NLR D28** | 0.08 | 0.21 | 0.22 | 0.14 | 0.03 | -0.08 | 0.18 | 0.61 | -0.04 | 0.07 | 0.15 | -0.08 | -0.05 | 0.01 | 0.43 | 1 |

CRP: C-reactive protein; NLR: Neutrophils to Lymphocytes Ratio; PD-L1 TC: Tumor PD-L1 expression;nivolumab: nivolumab level at day 28; VEGFA : Vascular Endothelial Growth Factor A. Concentrations were compared using a Spearman test. Two factors are interrelated if spearmans correlation coefficient was above 0.5 (positive correlation) or under -0.5 (negative correlation).
