# Supplementary figures and images for: Predictive Value of Soluble PD-1, PD-L1, VEGFA, CD40 Ligand and CD44 for Nivolumab Therapy in Advanced Non-Small Cell Lung Cancer: A Case-Control Study
Source: Cancers (Basel). 2020 Feb 18;12(2):473. doi: 10.3390/cancers12020473 (PMC7072584; doi:10.3390/cancers12020473)

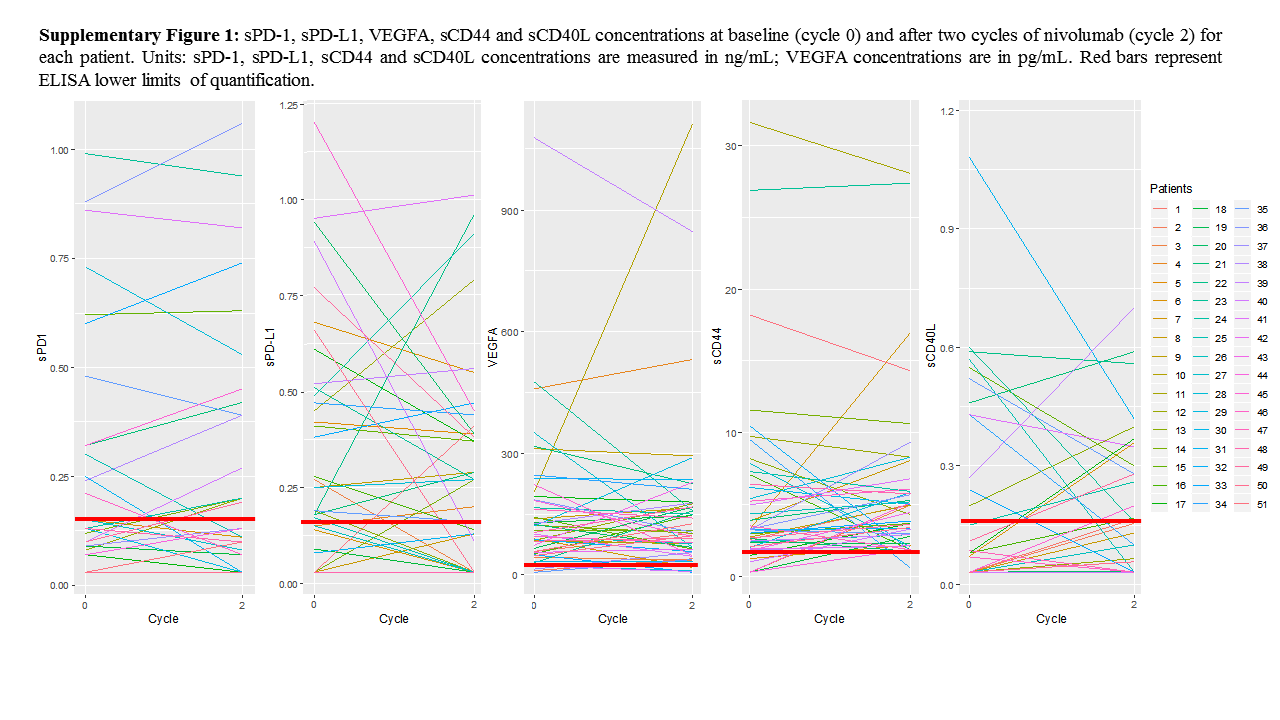

Supplement: Supplementary file 1 [file cancers-12-00473-s001.zip › cancers-707774-supplementary figures and tables/Supp Fig 1.PNG]

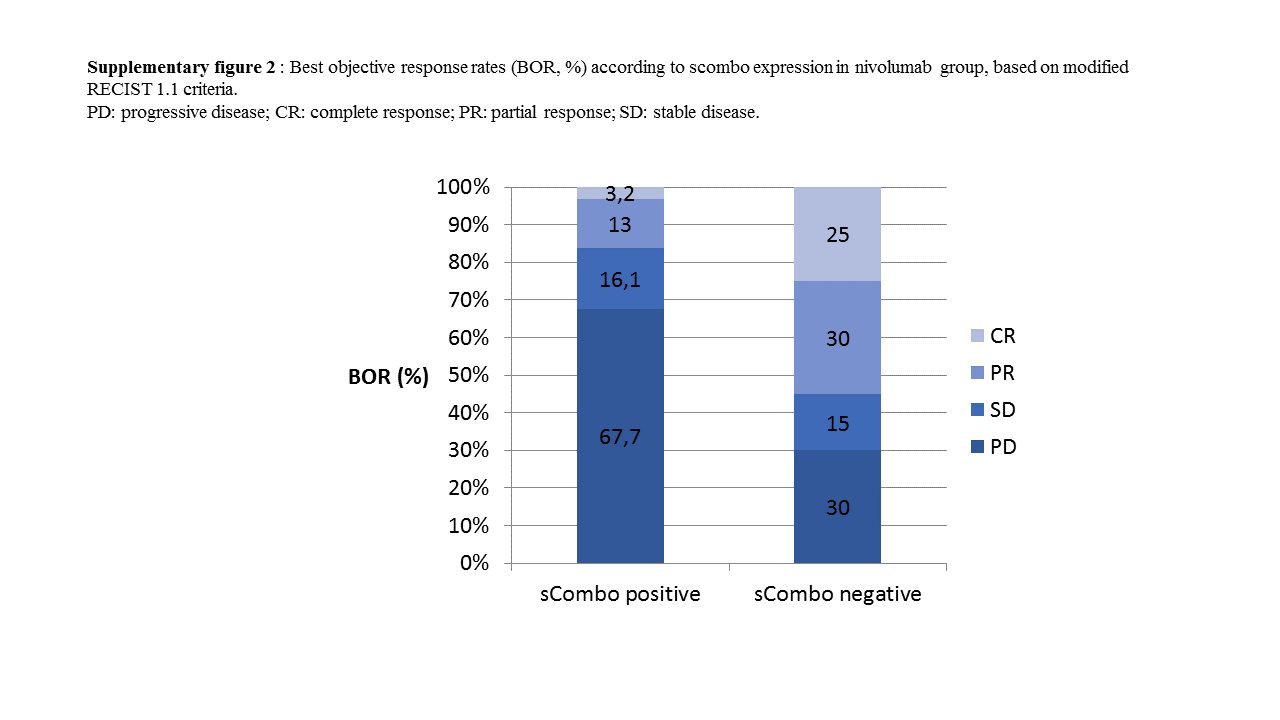

Supplement: Supplementary file 1 [file cancers-12-00473-s001.zip › cancers-707774-supplementary figures and tables/Supp Fig 2.PNG]
